# Supplementary figures and images for: Impact of an angulated aorto-septal relationship on cardio-cerebrovascular outcomes in patients undergoing hemodialysis
Source: PLoS One. 2024 Feb 23;19(2):e0298637. doi: 10.1371/journal.pone.0298637 (PMC10890729; doi:10.1371/journal.pone.0298637)

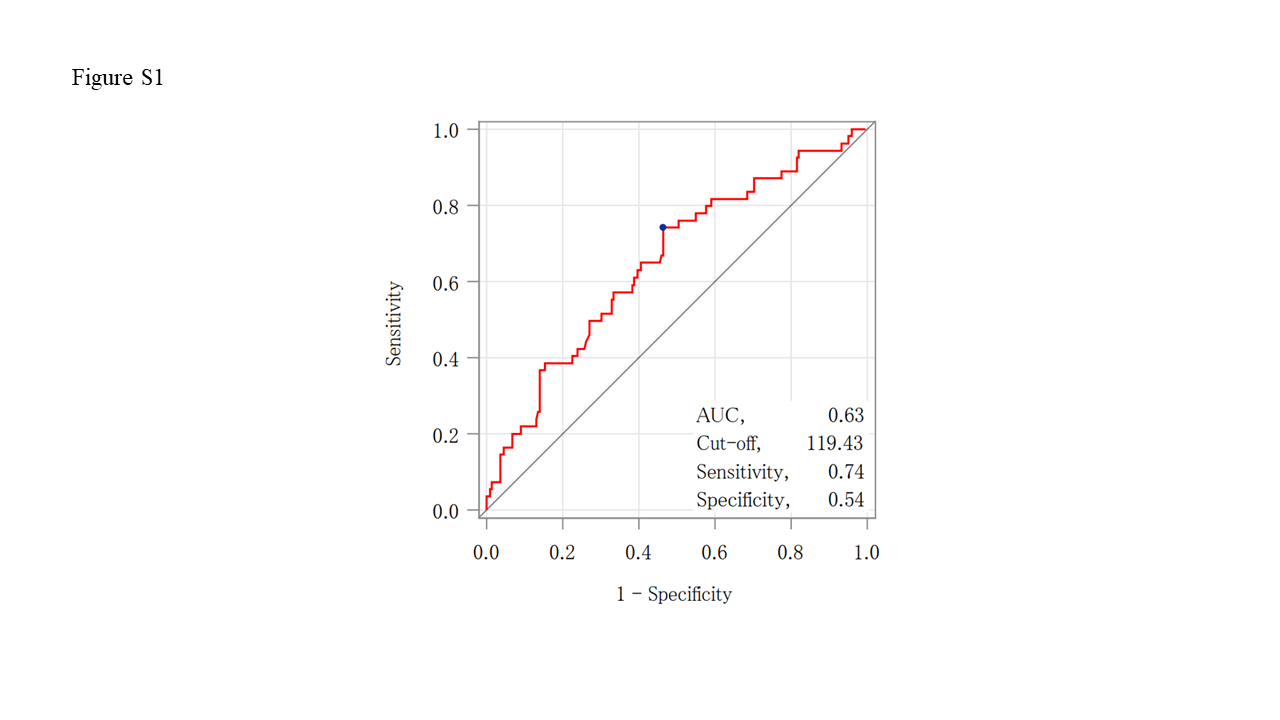

Supplement: S1 Fig — The optimal cut-off value was 119.4 degrees as determined by Youden’s index. The sensitivity and specificity of the cut-off value were 0.48 and 0.73, respectively. (TIF) [file pone.0298637.s001.tif]
