# Supplementary material for: Impact of an angulated aorto-septal relationship on cardio-cerebrovascular outcomes in patients undergoing hemodialysis
Source: PLoS One. 2024 Feb 23;19(2):e0298637. doi: 10.1371/journal.pone.0298637 (PMC10890729; doi:10.1371/journal.pone.0298637)
Supplement: S1 Table — A-I. Additional Cox proportional hazards analyses. (DOCX) [file pone.0298637.s002.docx]

| Table S1A. Additional Cox proportional hazards analyses | | | |  |  |  |  |  |
| --- | --- | --- | --- | --- | --- | --- | --- | --- |
| **Characteristic** | **Univariable analyses** | | |  | **Multivariable analyses** | | |  |
|  | **HR** | **95% CI** | **P value** |  | **HR** | **95% CI** | **P value** |  |
| Echocardiography |  |  |  |  |  |  |  |  |
| LVEF, per 10-% increase | 0.62 | 0.48-0.75 | < 0.001 |  | 0.65 | 0.52-0.82 | < 0.001 |  |
| LV mass index, per 10-g/m^2^ increase | 1.18 | 1.10-1.28 | < 0.001 |  | 1.15 | 1.06-1.25 | 0.001 |  |
| ASA, per 10-degree increase | 0.64 | 0.51-0.81 | < 0.001 |  | 0.74 | 0.58-0.95 | 0.017 |  |
| AV calcification score | 1.20 | 1.10-1.32 | < 0.001 |  | 1.11 | 1.00-1.22 | 0.043 |  |
| MV calcification score | 1.48 | 1.20-1.83 | < 0.001 |  | 1.21 | 0.94-1.55 | 0.14 |  |
| Age, per 10-year increase | 1.59 | 1.25-2.01 | < 0.001 |  | 1.39 | 1.04-1.85 | 0.027 |  |
|  |  |  |  |  |  |  |  |  |
| Table S1B. | | | |  |  |  |  |  |
| **Characteristic** | **Univariable analyses** | | |  | **Multivariable analyses** | | |  |
|  | **HR** | **95% CI** | **P value** |  | **HR** | **95% CI** | **P value** |  |
| Echocardiography |  |  |  |  |  |  |  |  |
| LVEF, per 10-% increase | 0.62 | 0.48-0.75 | < 0.001 |  | 0.68 | 0.54-0.85 | 0.001 |  |
| LV mass index, per 10-g/m2 increase | 1.18 | 1.10-1.28 | < 0.001 |  | 1.14 | 1.06-1.24 | 0.001 |  |
| ASA, per 10-degree increase | 0.64 | 0.51-0.81 | < 0.001 |  | 0.69 | 0.54-0.88 | 0.003 |  |
| AV calcification score | 1.20 | 1.10-1.32 | < 0.001 |  | 1.15 | 1.04-1.26 | 0.005 |  |
| MV calcification score | 1.48 | 1.20-1.83 | < 0.001 |  | 1.28 | 0.99-1.66 | 0.065 |  |
| Male, vs female | 1.04 | 0.99-1.10 | 0.16 |  | 1.15 | 0.61-2.18 | 0.66 |  |
|  |  |  |  |  |  |  |  |  |
| Table S1C. |  |  |  |  |  |  |  |  |
| **Characteristic** | **Univariable analyses** | | |  | **Multivariable analyses** | | |  |
|  | **HR** | **95% CI** | **P value** |  | **HR** | **95% CI** | **P value** |  |
| Echocardiography |  |  |  |  |  |  |  |  |
| LVEF, per 10-% increase | 0.62 | 0.48-0.75 | < 0.001 |  | 0.68 | 0.54-0.86 | 0.001 |  |
| LV mass index, per 10-g/m^2^ increase | 1.18 | 1.10-1.28 | < 0.001 |  | 1.15 | 1.06-1.25 | 0.001 |  |
| ASA, per 10-degree increase | 0.64 | 0.51-0.81 | < 0.001 |  | 0.68 | 0.53-0.88 | 0.003 |  |
| AV calcification score | 1.20 | 1.10-1.32 | < 0.001 |  | 1.16 | 1.05-1.27 | 0.003 |  |
| MV calcification score | 1.48 | 1.20-1.83 | < 0.001 |  | 1.34 | 1.03-1.74 | 0.027 |  |
| BMI, kg/m**^2^** | 1.04 | 0.99-1.10 | 0.16 |  | 1.06 | 1.01-1.13 | 0.032 |  |
|  |  |  |  |  |  |  |  |  |
| Table S1D. |  |  |  |  |  |  |  |  |
| **Characteristic** | **Univariable analyses** | | |  | **Multivariable analyses** | | |  |
|  | **HR** | **95% CI** | **P value** |  | **HR** | **95% CI** | **P value** |  |
| Echocardiography |  |  |  |  |  |  |  |  |
| LVEF, per 10-% increase | 0.62 | 0.48-0.75 | < 0.001 |  | 0.70 | 0.56-0.88 | 0.002 |  |
| LV mass index, per 10-g/m^2^ increase | 1.18 | 1.10-1.28 | < 0.001 |  | 1.15 | 1.06-1.25 | 0.001 |  |
| ASA, per 10-degree increase | 0.64 | 0.51-0.81 | < 0.001 |  | 0.68 | 0.53-0.87 | 0.002 |  |
| AV calcification score | 1.20 | 1.10-1.32 | < 0.001 |  | 1.15 | 1.04-1.26 | 0.005 |  |
| MV calcification score | 1.48 | 1.20-1.83 | < 0.001 |  | 1.28 | 0.99-1.66 | 0.056 |  |
| Cardiovascular risk factors |  |  |  |  |  |  |  |  |
| Diabetes mellitus | 1.81 | 1.06-3.01 | 0.030 |  | 1.70 | 0.98-1.94 | 0.059 |  |
|  |  |  |  |  |  |  |  |  |
| Table S1E. |  |  |  |  |  |  |  |  |
| **Characteristic** | **Univariable analyses** | | |  | **Multivariable analyses** | | |  |
|  | **HR** | **95% CI** | **P value** |  | **HR** | **95% CI** | **P value** |  |
| Echocardiography |  |  |  |  |  |  |  |  |
| LVEF, per 10-% increase | 0.62 | 0.48-0.75 | < 0.001 |  | 0.69 | 0.55-0.87 | 0.002 |  |
| LV mass index, per 10-g/m^2^ increase | 1.18 | 1.10-1.28 | < 0.001 |  | 1.14 | 1.05-1.24 | 0.002 |  |
| ASA, per 10-degree increase | 0.64 | 0.51-0.81 | < 0.001 |  | 0.69 | 0.54-0.89 | 0.004 |  |
| AV calcification score | 1.20 | 1.10-1.32 | < 0.001 |  | 1.13 | 1.03-1.25 | 0.011 |  |
| MV calcification score | 1.48 | 1.20-1.83 | < 0.001 |  | 1.27 | 0.99-1.64 | 0.060 |  |
| History of cardio-cerebrovascular disease | 2.42 | 1.38-4.23 | 0.002 |  | 1.51 | 0.84-2.71 | 0.17 |  |
|  |  |  |  |  |  |  |  |  |
| Table S1F. |  |  |  |  |  |  |  |  |
| **Characteristic** | **Univariable analyses** | | |  | **Multivariable analyses** | | |  |
|  | **HR** | **95% CI** | **P value** |  | **HR** | **95% CI** | **P value** |  |
| Echocardiography |  |  |  |  |  |  |  |  |
| LVEF, per 10-% increase | 0.62 | 0.48-0.75 | < 0.001 |  | 0.67 | 0.53-0.84 | 0.001 |  |
| LV mass index, per 10-g/m^2^ increase | 1.18 | 1.10-1.28 | < 0.001 |  | 1.15 | 1.06-1.25 | 0.001 |  |
| ASA, per 10-degree increase | 0.64 | 0.51-0.81 | < 0.001 |  | 0.70 | 0.54-0.89 | 0.004 |  |
| AV calcification score | 1.20 | 1.10-1.32 | < 0.001 |  | 1.14 | 1.04-1.26 | 0.007 |  |
| MV calcification score | 1.48 | 1.20-1.83 | < 0.001 |  | 1.21 | 0.94-1.54 | 0.14 |  |
| Laboratory measurements |  |  |  |  |  |  |  |  |
| Albumin, g/dL | 0.44 | 0.23-0.85 | 0.015 |  | 0.49 | 0.23-1.05 | 0.066 |  |
|  |  |  |  |  |  |  |  |  |
| Table S1G. |  |  |  |  |  |  |  |  |
| **Characteristic** | **Univariable analyses** | | |  | **Multivariable analyses** | | |  |
|  | **HR** | **95% CI** | **P value** |  | **HR** | **95% CI** | **P value** |  |
| Echocardiography |  |  |  |  |  |  |  |  |
| LVEF, per 10-% increase | 0.62 | 0.48-0.75 | < 0.001 |  | 0.66 | 0.52-0.83 | < 0.001 |  |
| LV mass index, per 10-g/m^2^ increase | 1.18 | 1.10-1.28 | < 0.001 |  | 1.13 | 1.04-1.23 | 0.003 |  |
| ASA, per 10-degree increase | 0.64 | 0.51-0.81 | < 0.001 |  | 0.68 | 0.53-0.87 | 0.002 |  |
| AV calcification score | 1.20 | 1.10-1.32 | < 0.001 |  | 1.14 | 1.04-1.26 | 0.008 |  |
| MV calcification score | 1.48 | 1.20-1.83 | < 0.001 |  | 1.27 | 0.99-1.63 | 0.061 |  |
| Laboratory measurements |  |  |  |  |  |  |  |  |
| Total bilirubin, per 0.1-mg/dL increase | 1.17 | 0.98-1.41 | 0.085 |  | 1.14 | 0.91-1.41 | 0.26 |  |
|  |  |  |  |  |  |  |  |  |
| Table S1H. |  |  |  |  |  |  |  |  |
| **Characteristic** | **Univariable analyses** | | |  | **Multivariable analyses** | | |  |
|  | **HR** | **95% CI** | **P value** |  | **HR** | **95% CI** | **P value** |  |
| Echocardiography |  |  |  |  |  |  |  |  |
| LVEF, per 10-% increase | 0.62 | 0.48-0.75 | < 0.001 |  | 0.68 | 0.54-0.85 | 0.001 |  |
| LV mass index, per 10-g/m^2^ increase | 1.18 | 1.10-1.28 | < 0.001 |  | 1.14 | 1.05-1.24 | 0.002 |  |
| ASA, per 10-degree increase | 0.64 | 0.51-0.81 | < 0.001 |  | 0.69 | 0.54-0.88 | 0.003 |  |
| AV calcification score | 1.20 | 1.10-1.32 | < 0.001 |  | 1.14 | 1.03-1.25 | 0.010 |  |
| MV calcification score | 1.48 | 1.20-1.83 | < 0.001 |  | 1.28 | 0.99-1.64 | 0.055 |  |
| Laboratory measurements |  |  |  |  |  |  |  |  |
| Serum sodium, mEq/L | 0.92 | 0.85-0.99 | 0.023 |  | 0.95 | 0.87-1.04 | 0.24 |  |
|  |  |  |  |  |  |  |  |  |
| Table S1I. |  |  |  |  |  |  |  |  |
| **Characteristic** | **Univariable analyses** | | |  | **Multivariable analyses** | | |  |
|  | **HR** | **95% CI** | **P value** |  | **HR** | **95% CI** | **P value** |  |
| Echocardiography |  |  |  |  |  |  |  |  |
| LVEF, per 10-% increase | 0.62 | 0.48-0.75 | < 0.001 |  | 0.72 | 0.58-0.91 | 0.005 |  |
| LV mass index, per 10-g/m^2^ increase | 1.18 | 1.10-1.28 | < 0.001 |  | 1.16 | 1.07-1.26 | 0.001 |  |
| ASA, per 10-degree increase | 0.64 | 0.51-0.81 | < 0.001 |  | 0.67 | 0.52-0.86 | 0.002 |  |
| AV calcification score | 1.20 | 1.10-1.32 | < 0.001 |  | 1.13 | 1.03-1.24 | 0.013 |  |
| MV calcification score | 1.48 | 1.20-1.83 | < 0.001 |  | 1.29 | 0.99-1.67 | 0.058 |  |
| Medication |  |  |  |  |  |  |  |  |
| Anti-platelet or anti-coagulation | 2.87 | 1.68-4.92 | < 0.001 |  | 2.36 | 1.35-4.11 | 0.002 |  |
| ASA, aorto-septal angle, the angle between the anterior wall of the aorta and the ventricular septal surface; AV, aortic valvular; MV mitral valvular. | | | | | | | |  |
|  |  |  |  |  |  |  |  |  |
|  |  |  |  |  |  |  |  |  |
|  |  |  |  |  |  |  |  |  |
|  |  |  |  |  |  |  |  |  |
