# Supplementary material for: Impact of an angulated aorto-septal relationship on cardio-cerebrovascular outcomes in patients undergoing hemodialysis
Source: PLoS One. 2024 Feb 23;19(2):e0298637. doi: 10.1371/journal.pone.0298637 (PMC10890729; doi:10.1371/journal.pone.0298637)
Supplement: S2 Table — A. Correlations between dialytic date-interval and echocardiographic parameters. B. Additional Cox proportional hazards analyses. (DOCX) [file pone.0298637.s003.docx]

| Table S2A. Correlations between dialytic date-interval and echocardiographic parameters | | |  |
| --- | --- | --- | --- |
|  | **R** | **P value** |  |
| LV ejection fraction, % | -0.07 | 0.26 |  |
| LV diastolic diameter, mm | 0.23 | < 0.001 |  |
| LV systolic diameter, mm | 0.18 | 0.002 |  |
| LV wall thickness, mm | -0.04 | 0.55 |  |
| LV mass index, g/m^2^ | 0.01 | 0.81 |  |
| Left arterial diameter, mm | 0.14 | 0.01 |  |
| E/E' | -0.02 | 0.70 |  |
| ASA | 0.12 | 0.045 |  |
| AVCS | -0.05 | 0.35 |  |
| MVCS | -0.10 | 0.079 |  |
| LV, left ventricular; E/E', ratio of the early diastolic transmitral flow velocity to mitral annular velocity; ASA, aorto-septal angle; AVCS, aortic valve calcification score; MVCS, mitral valve calcification score. | | |  |

| Table S2B. Additional Cox proportional hazards analyses | | | |  |  | | |  | |  | |  |
| --- | --- | --- | --- | --- | --- | --- | --- | --- | --- | --- | --- | --- |
| **Characteristic** | **Univariable analyses** | | | | |  | **Multivariable analyses** | | | | | |
|  | **HR** | **95% CI** | **P value** | | |  | **HR** | | **95% CI** | | **P value** | |
| Echocardiography |  |  |  | | |  |  | |  | |  | |
| LVEF, per 10-% increase | 0.62 | 0.48-0.75 | < 0.001 | | |  | 0.67 | | 0.53-0.85 | | 0.001 | |
| LV mass index, per 10-g/m^2^ increase | 1.18 | 1.10-1.28 | < 0.001 | | |  | 1.14 | | 1.05-1.24 | | 0.001 | |
| ASA, per 10-degree increase | 0.64 | 0.51-0.81 | < 0.001 | | |  | 0.69 | | 0.54-0.88 | | 0.003 | |
| AV calcification score | 1.20 | 1.10-1.32 | < 0.001 | | |  | 1.15 | | 1.04-1.26 | | 0.005 | |
| MV calcification score | 1.48 | 1.20-1.83 | < 0.001 | | |  | 1.26 | | 0.98-1.61 | | 0.074 | |
| Dialytic date-interval, days | 0.97 | 0.72-1.32 | 0.84 | | |  | 1.01 | | 0.75-1.37 | | 0.93 | |
| ASA, aorto-septal angle, the angle between the anterior wall of the aorta and the ventricular septal surface; AV, aortic valvular; MV, mitral valvular. | | | | | | | | | | | | |
