# Supplementary material for: Impact of an angulated aorto-septal relationship on cardio-cerebrovascular outcomes in patients undergoing hemodialysis
Source: PLoS One. 2024 Feb 23;19(2):e0298637. doi: 10.1371/journal.pone.0298637 (PMC10890729; doi:10.1371/journal.pone.0298637)
Supplement: S3 Table — A. Aortic valve and mitral valve calcification scores for patients with moderate or greater aortic valve stenosis. B. Additional Cox proportional hazards analyses. (DOCX) [file pone.0298637.s004.docx]

| Table S3A. Aortic valve (AV) and mitral valve (MV) calcification scores for patients with moderate or greater aortic valve stenosis | | | | |  |
| --- | --- | --- | --- | --- | --- |
| AV stenosis ≥ moderate (n=4) | Age | Sex | AV calcification score | MV calcification score |  |
| Patient No.1 | 65 | Male | 8 | 1 |  |
| Patient No.2 | 77 | Male | 8 | 2 |  |
| Patient No.3 | 81 | Female | 8 | 3 |  |
| Patient No.4 | 66 | Male | 9 | 1 |  |
|  | | | | |  |
|  |  |  |  |  |  |
|  |  |  |  |  |  |

| Table S3B. Additional Cox proportional hazards analyses | | | |  |  | |  | |  | |  |
| --- | --- | --- | --- | --- | --- | --- | --- | --- | --- | --- | --- |
| **Characteristic** | **Univariable analyses** | | | |  | **Multivariable analyses** | | | | | |
|  | **HR** | **95% CI** | **P value** | |  | **HR** | | **95% CI** | | **P value** | |
| Echocardiography |  |  |  | |  |  | |  | |  | |
| LVEF, per 10-% increase | 0.62 | 0.48-0.75 | < 0.001 | |  | 0.67 | | 0.53-0.84 | | 0.001 | |
| LV mass index, per 10-g/m^2^ increase | 1.18 | 1.10-1.28 | < 0.001 | |  | 1.14 | | 1.05-1.24 | | 0.001 | |
| ASA, per 10-degree increase | 0.64 | 0.51-0.81 | < 0.001 | |  | 0.69 | | 0.54-0.88 | | 0.003 | |
| AV calcification score | 1.20 | 1.10-1.32 | < 0.001 | |  | 1.15 | | 1.04-1.28 | | 0.007 | |
| MV calcification score | 1.48 | 1.20-1.83 | < 0.001 | |  | 1.25 | | 0.98-1.60 | | 0.072 | |
| Aortic valve stenosis ≥ moderate | 4.39 | 1.07-18.06 | 0.041 | |  | 0.86 | | 0.19-3.95 | | 0.85 | |
| ASA, aorto-septal angle, the angle between the anterior wall of the aorta and the ventricular septal surface; AV, aortic valvular; MV, mitral valvular. | | | | | | | | | | | |
