# Supplementary material for: Impact of an angulated aorto-septal relationship on cardio-cerebrovascular outcomes in patients undergoing hemodialysis
Source: PLoS One. 2024 Feb 23;19(2):e0298637. doi: 10.1371/journal.pone.0298637 (PMC10890729; doi:10.1371/journal.pone.0298637)
Supplement: Attachment — Submitted filename: REVIEW COMMENTS.docx [file pone.0298637.s006.docx]

**Manuscript Number:** PONE-D-23-33216
**Manuscript Title:** Impact of Sigmoid Septum on Cardio-cerebrovascular Outcomes in Patients Undergoing Hemodialysis

**Review comments.**

The manuscript **is fit for publication** fulfilling the journals criteria. As a minor revision a comment could be added in the discussion regarding the presence of Q waves on the ECG and their correlation with the presence of sigmoid septum. Dialysis patients are a special population with increased incidence of ventricular hypertrophy or cardiovascular diseases. So a comment could be added regarding the differentil diagnosis of the presence of Q-waves on the ECG, based on an other article entitled ‘’ Correlation between sigmoid interventricular septum angle and presence of Q waves on the electrocardiogram’’.
