# Supplementary material for: Impact of an angulated aorto-septal relationship on cardio-cerebrovascular outcomes in patients undergoing hemodialysis
Source: PLoS One. 2024 Feb 23;19(2):e0298637. doi: 10.1371/journal.pone.0298637 (PMC10890729; doi:10.1371/journal.pone.0298637)
Supplement: Attachment — Submitted filename: Response_to_Reviewers_2nd_revision.docx [file pone.0298637.s008.docx]

**Reply to the Editor**

We want to thank the editor for the warm response. With the comments, we were able to update our knowledge for the research analysis.

**Editor’s comment_1)**
According to Figure 3, censoring appears to have occurred in many cases after 36 months. It may be inappropriate to assess endpoints beyond this 36-month period.

**Reply)**

We re-assessed the endpoints within 36 months. The number for the primary endpoint decreased from 56 to 54. We updated the baseline characteristics (Table 1), Cox proportional hazard analyses (Table 2, Table S1A-S1I, Table S2B, and S3B), and Kaplan-Meier analysis using the new endpoint information, and we verified that there was no need to change the conclusions. Before the correction, the Kaplan-Meier curve was drawn as 30 days per month. After the correction, the Kaplan-Meier curve was 365 days per year (182 days per 6 months), resulting in a slight change in the number at risk.

Moreover, at the individual's request, the sentence "H.H. reviewed and supervised the statistical analyses of the current study" has been removed. H.H. strictly supervised our statistical analyses in the original and revised version of the manuscript, but his policy is not to make this statement in the manuscript.

**<Page 3, lines 11-19>**

**Before revised:**

The primary endpoint was observed in 56 patients during the observational period (median 1183 days). Multivariable Cox proportional hazards analyses identified left ventricular ejection fraction (per 10% increase: hazard ratio [HR] 0.68; 95% confidential interval [CI] 0.54-0.85, P = 0.001), left ventricular mass index (per 10 g/m^2^ increase: HR 1.13; 95% CI 1.05-1.23, P = 0.002), ASA (per 10 degree increase: HR 0.71; 95% CI 0.56-0.91; P = 0.006), and aortic valve calcification score (HR 1.16; 95% CI 1.06-1.28, P = 0.002) as independent determinants of the primary endpoint. Kaplan-Meier analysis showed a higher incidence of the primary endpoint in patients with ASA <119.4 degrees than those with ASA ≥119.4 degrees (Log-rank P = 0.001).

**After revised:**

The primary endpoint was observed in 54 patients during the observational period (median 1095 days). Multivariable Cox proportional hazards analyses identified left ventricular ejection fraction (per 10% increase: hazard ratio [HR] 0.67; 95% confidential interval [CI] 0.53-0.84, P = 0.001), left ventricular mass index (per 10 g/m^2^ increase: HR 1.14; 95% CI 1.05-1.24, P = 0.001), ASA (per 10 degree increase: HR 0.69; 95% CI 0.54-0.88; P = 0.003), and aortic valve calcification score (HR 1.15; 95% CI 1.04-1.26, P = 0.005) as independent determinants of the primary endpoint. Kaplan-Meier analysis showed a higher incidence of the primary endpoint in patients with ASA <119.4 degrees than those with ASA ≥119.4 degrees (Log-rank P < 0.001).

**<Page 8, lines 24-25>**

**Before revised:**

As the patients’ prognoses were retrospectively verified between September and December 2021, the primary endpoint observed in the current study included the events observed after 36 months.

**After revised:**

In this retrospective study, patient prognosis was evaluated up to 36 months.

**<Page 12, lines 3-4>**

**Before revised:**

The primary endpoint was observed in 56 patients during the observational period, which was a median 1183 days (interquartile range, 908-1196 days).

**After revised:**

The primary endpoint was observed in 54 patients during the observational period, which was a median 1095 days (interquartile range, 908-1095 days).

**<Page 12, lines 7-12>**

**Before revised:**

Multivariable Cox proportional hazards analyses identified LVEF (per 10% increase: hazard ratio [HR] 0.68; 95% confidential interval [CI] 0.54-0.85; P = 0.001), LV mass index (per 10 g/m^2^ increase: HR 1.13; 95% CI 1.05-1.23; P = 0.002), ASA (per 10 degree increase; HR 0.71; 95% CI 0.56-0.91; P = 0.006), and AVCS (HR 1.16; 95% CI 1.06-1.28; P = 0.002) as independent determinants of the primary endpoint (Table 2).

**After revised:**

Multivariable Cox proportional hazards analyses identified LVEF (per 10% increase: hazard ratio [HR] 0.67; 95% confidential interval [CI] 0.53-0.84; P = 0.001), LV mass index (per 10 g/m^2^ increase: HR 1.14; 95% CI 1.05-1.24; P = 0.001), ASA (per 10 degree increase; HR 0.69; 95% CI 0.54-0.88; P = 0.003), and AVCS (HR 1.15; 95% CI 1.04-1.26; P = 0.005) as independent determinants of the primary endpoint (Table 2).

**<Page 9, lines 2>**

**Before revised:**

H.H. reviewed and supervised the statistical analyses of the current study.

**After revised:**

Deleted.

**Reply to the Reviewer #1**

We appreciate the reviewer's careful, kind, and sincere review. The constructive comments and scientific points were crucial for our future work.

**Reviewer’s comment_1)**
Minor comments)
\# It seems that the author’s group had published multiple papers focusing on the “sigmoid septum”. If their previous methodologies used to evaluate the “sigmoid septum” were similar to the one utilized in this study, it would be better to explain that “angulated aorto-septal relationship” they used in this study is the same phenotypes that they referred to as the “sigmoid septum” in their previous studies, and the reason they changed the terminology. I believe that the authors now clearly appreciate the difference between these two concepts.

**Reply)**

We thank the reviewer for the scientifically important comment. We must mention and explain why AVA was used as the representative of aortic wedging due to aortic elongation, which was different from the representative of sigmoid septum used in our previous investigations. The reason was our updated understanding of the difference between sigmoid septum and aortic wedging (because of the reviewer’s valuable comments).

We added sentences explaining this in the Discussion section.

**<Page 21, lines 12-17>**

**Before revised:**

None.

**After revised:**

In a previous study, we used ASA as a representative parameter of sigmoid septum. However, as we updated our knowledge with the latest research, we came to appreciate these two different morphological concepts; it is correct to use ASA to indicate aortic wedging due to aortic elongation. Aortic wedging and sigmoid septum look similar but, as mentioned above, these two concepts must be distinguished regarding different substrates and likely different clinical impacts.

**Reviewer’s comment_2)**
\# “Sometimes variously termed subaortic ventricular septal bulge and discrete upper septal hypertrophy, sigmoid septum is associated with age [27-29], with atherosclerosis, thickening of the aortic or mitral valve, and hypertension considered to contribute to its presence [30-32].” This sentence does not make sense, I mean overall English of this paper needs professional English editing service before resubmission.

**Reply)**

We apologize for our insufficient English. Our manuscript underwent editing by a professional native English speaker twice, before the first submission and before submission of the revision. However, as the reviewer was concerned, we changed some sentences after the English editing. The changed sentences were edited by a professional English editor prior to this resubmission.

**<Page 21, lines 1-4>**

**Before revised:**

Sometimes variously termed subaortic ventricular septal bulge and discrete upper septal hypertrophy, sigmoid septum is associated with age, with atherosclerosis, thickening of the aortic or mitral valve, and hypertension considered to contribute to its presence.

**After revised:**

Sigmoid septum, which is sometimes referred to as subaortic ventricular septal bulge or discrete upper septal hypertrophy, is associated with age. In addition, atherosclerosis, thickening of the aortic or mitral valve, and hypertension are considered to contribute to the presence of sigmoid septum.

**Reviewer’s comment_3)**
\# “Initially, sigmoid septum was defined as the base of the ventricular septum protruding toward the LV cavity [26].” I think the original study that defined the “sigmoid septum” was the one by Goor D, et al. (Am J Roentgenol Radium Ther Nucl Med. 1969;107:366–376.)

**Reply)**

We appreciate the reviewer pointing this out and apologize for the confusing description. We used reference number [26] to cite the sigmoid septum in the "Response to Reviewer" file according to the appearance order in the "Response to Reviewer," and we used reference number [39] to cite the same reference in the manuscript. This reference was the same as the one provided by the reviewer.

**Reviewer’s comment_4)**
\# “Interestingly, an angulated relationship between the ventricular septum and ascending aorta did not correlate with the extent of septal bulge in prior research using computed tomography [22],” I am afraid citation of this sentence might be incorrect. This sentence seems to represent the findings by Tsuda et al (Echocardiography. 2022;39:248-259.).

**Reply)**

We want to thank the reviewer for providing an accurate citation. We verified that the findings by Tsuda et al. are more suitable than ours (Mori et al. J Anat. 2017;231(1):110-20.) and replaced the reference.
